# High-Titanium Slag Concrete with Multiscale Pores: Enhanced Explosive Stress Wave Dissipation for Underground Defense

**DOI:** 10.3390/ma18194609

**Published:** 2025-10-05

**Authors:** Weiting Gao, Meng Wang, Jinshan Sun

**Affiliations:** 1 State Key Laboratory of Precision Blasting, Jianghan University, Wuhan 430056, China; 2 Hubei Key Laboratory of Blasting Engineering, Jianghan University, Wuhan 430056, China; 3 State Key Laboratory of Intelligent Construction and Healthy Operation and Maintenance of Deep Underground Engineering, College of Architecture and Environment, Sichuan University, Chengdu 610065, China; gaoweitingscu@126.com

**Keywords:** high-titanium slag concrete, multiscale pores, stress wave attenuation

## Abstract

Balancing stress wave attenuation with structural integrity is recognized as a critical challenge for protective materials in underground defense systems. A novel high-titanium slag (HTS) concrete featuring multiscale pores is proposed to address this dilemma. Large-particle porous HTS aggregates are embedded into cement mortar, enabling mechanical robustness comparable to conventional concrete alongside significant stress wave dissipation. Wave scattering and gas–solid interfacial reflections are induced by the multiscale pore architecture, effectively attenuating energy propagation. A dense interface transition zone between HTS aggregates and the cement mortar is confirmed through microscopic characterization, ensuring structural coherence. Wave attenuation is revealed by Split Hopkinson Pressure Bar tests to primarily originate from pore-driven reflections rather than impedance mismatch. A groundbreaking strategy is offered for designing blast-resistant materials that harmonize dynamic energy dissipation with structural durability, advancing the development of resilient underground infrastructure.

## 1. Introduction

In the three-dimensional offensive-defensive framework of modern warfare, underground defense structures are the pivotal carriers of strategic deterrence. The integration of earth-penetrating weapons with high-explosive warheads renders the propagation and fracturing effects of explosive stress waves the foremost peril confronting underground engineering [1]. When shockwaves reflect and accumulate repeatedly within a confined space, the spalling failure of concrete structures typically commences from the internal micro-crack network of the material [2]. The uniqueness of this failure mechanism lies in the coupling between the energy dissipation characteristics of stress waves and the mesoscopic structure of protective materials [3]. The propagation of stress waves in concrete or rock media displays pronounced non-linear traits [4,5,6]. During the initial impact phase, compressive longitudinal waves prevail. In the subsequent reflection phases, the combined action of shear and surface waves generates a multi-directional tensile stress field, which is the mechanical origin of concrete spalling failure.

To enhance the wave-regulating ability of concrete, the engineering community has successively developed two main technical approaches. The first approach involves the introduction of metal-based porous materials, aiming to achieve efficient energy absorption through plastic deformation [7,8], such as foamed aluminum, where the energy dissipation increases by 13.8% [9]. However, the difference in thermal expansion coefficients between the metal-based materials and the concrete leads to interfacial delamination under temperature-change conditions. The second approach turns to the optimization of porous concrete [10,11,12]. Although foamed concrete can significantly attenuate the peak pressure of stress waves, its compressive strength is often less than 10 MPa [13]. These explorations reveal a fundamental contradiction: any improvement in a single performance dimension comes at the cost of sacrificing the material’s systemic nature. The breakthrough in protective materials must return to the essence of the concrete system and achieve a performance leap while maintaining its engineering genes.

Recent investigations have shown that high-titanium slag, a by-product of titanium extraction, can serve as a high-performance aggregate in cementitious composites [14,15]. Its unique mineralogy—dominated by perovskite, rutile, and silicate glass—confers substantial compressive strength, often exceeding 60 MPa in standalone form [16]. Notably, HTS concrete not only retains but can enhance composite strength: one study reported that ultra-high-performance concrete (UHPC) using titaniferous slag sand achieved a higher 28-day compressive strength compared to conventional quartz sand concrete [17]. This improvement is attributed to a denser interfacial transition zone (ITZ) and superior particle interlock [18]. Therefore, in contrast to typical porous fillers, HTS simultaneously delivers energy-absorption potential and high static strength—making it ideal for protective concrete applications.

High-titanium slag aggregates commonly contain a significant amount of internal pores, with diameters typically ranging from 0.5 to 3 mm. These cavities, mostly elliptical and either closed or partially connected, are formed during high-temperature cooling through gas release and phase separation [19]. As a result, HTS aggregates inherently possess a multi-scale porous system when used as coarse aggregates in concrete. Existing studies have demonstrated that pore structures play a critical role in stress-wave propagation, inducing wave scattering, mode conversion, and localized reflection [20,21,22]. However, current research has primarily focused on foamed concrete or artificially embedded cavities, while the behavior of naturally porous aggregates in regulating wave dynamics remains insufficiently explored [23,24,25,26,27,28]. In particular, how pore distribution and local impedance mismatch within high-strength, heterogeneous aggregates influence stress-wave attenuation—without compromising structural integrity—remains unclear. This gap constitutes one of the key scientific questions addressed in the present study.

In this paper, a novel cement-based composite with large-particle porous high-titanium slag (HTS) as functional aggregate, which solves the key problem of simultaneous coordination between dynamic crack resistance and stress wave attenuation performance in protective materials, is proposed. At the material design level, a multi-scale pore system is formed through gradation optimization of large-grained slag, and stress wave scattering attenuation is induced by the pore network. Compressive strength testing of the new material is conducted. Meanwhile, wave propagation is verified by the Split Hopkinson Pressure Bar (SHPB), and the effectiveness of the material in stress wave attenuation is tested. The experimental validity and the mechanical performance of HTS were further verified through industrial CT scanning and SEM analysis. The finite difference software AUTODYN (V-19.2) was employed to construct a numerical model of HTS concrete, providing deeper insight into the mechanisms of stress-wave attenuation.

## 2. Materials and Methods

### 2.1. Preparation of Experimental Materials

High-titanium slag (HTS) is a hard, porous material produced by cooling blast-furnace slag generated during the smelting of vanadium–titanium magnetite at Panzhihua Iron and Steel Co., Ltd. (Panzhihua, China). Prior studies have shown that HTS can be used as a coarse aggregate in concrete; HTS concrete attains a strength class of C30 or higher and, with appropriate mix design, can reach C80 [29].

**Figure 1:**
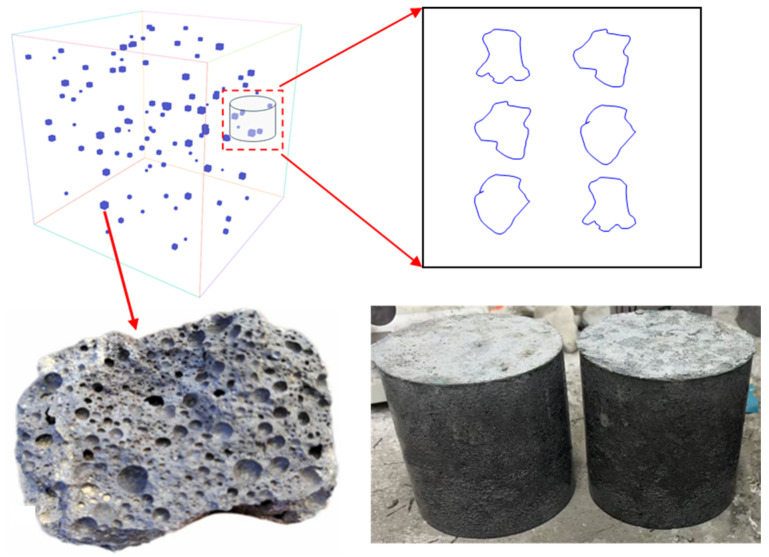
Physical model extracted for investigating the stress-wave absorption of high-titanium slag (HTS).

As illustrated in Figure 1, HTS serves as the coarse aggregate in HTS concrete, the shape of HTS is irregular, and the cross-sections shown are approximate results extracted from CT scans used in this study. During casting, the internal pore structure of the HTS is preserved, and this pore system exerts a pronounced attenuating effect on shock waves. However, directly tracking how stress waves evolve as they propagate through HTS concrete is challenging. Therefore, in this study, we magnify the internal HTS coarse-aggregate structure by directly casting large, block-like HTS particles into the cement mortar, thereby enabling investigation of the mechanism by which HTS concrete absorbs stress-wave energy. The pores in high-titanium slag were formed by water-quenching of the blast-furnace slag produced during vanadium–titanium magnetite smelting. The composition of HTS is shown in Table 1.

To more precisely characterize the titanium slag, porosity was measured by the water-saturation method (Equation (1)). The titanium slag samples were first oven-dried and weighed to obtain their dry mass ($W_{dry}$). The volume of each sample was then measured using the water displacement method. After fully immersing the samples in water and allowing them to reach saturation, their saturated mass ${(W}_{sat})$ was recorded. The porosity was calculated by dividing the difference between the saturated and dry weights by the product of water density and slag volume. Multiple tests showed that the porosity of titanium slag ranged from 37.9% to 44%, with an average value of 41.2%. The density of the titanium slag was calculated by dividing the dry mass by its volume, yielding a value of 2739 $Kg/m^{3}$.

(1) $$\phi =\frac{W_{sat}-W_{dry}}{\rho_{water}\times V}$$

Two groups were set up in the experiment, each with three specimens. One was the experimental group, which was mixed with uncrushed large particle titanium slag in cement mortar, named the TC group. One was the control group, which used pure cement mortar without slag and was called the CM group. The specimen is a cylinder of Φ100 mm × 100 mm. The CM group’s material mix ratio (by weight) is cement/sand/water/water reducer = 1:2.04:0.33:0.01, using P·O42.5R ordinary Portland cement as the cementitious binder and natural river sand with a particle size of 0.2–0.5 mm as the aggregate. To optimize slurry performance, a polycarboxylate superplasticizer was added to enhance the fluidity of the cement mortar, ensuring good workability for construction. Ordinary tap water meeting relevant standards was used, guaranteeing no adverse effects on the material properties.

**Figure 2:**
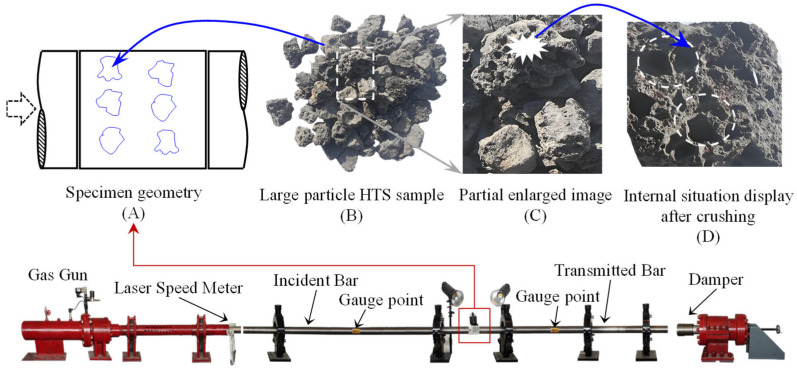
The schematic diagram of the experimental setup and specimen. (**A**) The sketch diagram of the specimen. (**B**) Large particle HTS sample. (**C**) Partial enlarged image of HTS. (**D**) Internal situation display after crushing.

The TC group is prepared by adding nine HTS particles to the CM group material. Sourced as a by-product from vanadium–titanium magnetite smelting, these slag particles serve as coarse aggregates characterized by a diameter distribution of 15–20 mm, as illustrated in Figure 2B. The magnified morphology of the coarse aggregate is depicted in Figure 2C. Following the selection and identification of representative aggregate particles, manual fragmentation using a hammer reveals their internal structure, as shown in Figure 2D, where a distinct porous architecture is observable within the particles. The SHPB (Laboratory of Impact Mechanics, Sichuan University, Chengdu, China) was used as a test device, as shown in Figure 2, with a diameter of 100 mm. Also, it is necessary to ensure that the specimen and the two bars are closely bonded to ensure stress wave propagation.

### 2.2. Split Hopkinson Pressure Bar (SHPB) Testing System

To obtain the stress wave transmission characteristics of the specimens, their integrity is maintained during the test to eliminate energy consumption caused by material fracture. Taking CM1 and TC1 as examples, stress wave monitoring data from the incident and transmission bars are presented in Figure 2. When stress waves interact with the specimen, reflection and transmission waves are generated at the interface due to the specimen’s wave impedance being lower than that of the testing equipment’s bars. The stress waves propagate through the specimen and eventually transfer into the transmission bar, with the monitoring data from the transmission bar representing the results after a series of reflections and transmissions within the system. To quantify the material’s influence on stress waves, transmissivity is defined and expressed by Equation (2).

(2) $$\alpha =\left|\frac{T_{max}}{I_{max}}\right|\times 100\%$$

where *T_max_* is the maximum value of the transmitted stress wave, and *I_max_* is the maximum value of the incident stress wave.

In addition to quantifying the reduction in stress-wave amplitude, it is essential to evaluate the decay of the energy transported by the wave. Within the SHPB apparatus, the energy carried by the incident and transmitted bars can be calculated by integrating their respective strain histories. The resulting energy loss after the wave traverses the specimen is obtained via Equation (3). We further define the energy-attenuation coefficient *β*, which is given by Equation (4).

(3) $$\left\{\begin{array}{c} W_{I}\left(t\right)=EAc\int_{0}^{\tau }\varepsilon_{I}^{2}(t)dt\\ W_{R}\left(t\right)=EAc\int_{0}^{\tau }\varepsilon_{R}^{2}(t)dt\\ W_{T}\left(t\right)=EAc\int_{0}^{\tau }\varepsilon_{T}^{2}(t)dt \end{array}\right.$$

(4) $$\beta =\frac{W_{I}-W_{R}-W_{T}}{WI}\times 100\%$$

where *E* represents the elastic modulus of the two bars in the SHPB device, *c* represents the wave velocity, *W_I_*, *W_R_*, *W_T_* correspond to the energies of the incident, reflected, and time-dependent transmitted waves, respectively. *β* is the energy-attenuation coefficient.

### 2.3. Industrial Computed Tomography (CT) and Scanning Electron Microscopy (SEM)

To visualize the three-dimensional pore architecture introduced by high-titanium slag (HTS) aggregates without damaging, industrial computed tomography was performed prior to the impact tests. Cylindrical concrete samples were scanned with an X-ray energy of 90 kV and 120 μA, yielding a voxel resolution of 80 µm. Because the peak amplitude of the incident stress wave used in the subsequent split Hopkinson pressure bar (SHPB) experiments was selected to remain below the elastic limit of the concrete, the CT data reliably represent the undamaged internal state. The reconstructed pore architecture serves as a direct geometric and parametric reference for subsequent numerical modeling.

To complement the volumetric CT observations with sub-micron detail, polished cross-sections extracted from the same mixes were examined by scanning electron microscopy. Specimens were dried under vacuum, impregnated with low-viscosity epoxy, diamond-polished to 1 µm, and sputter-coated with a 10 nm Au–Pd layer to ensure electrical conductivity. Back-scattered electron (BSE) images were acquired at 15 kV and 10 mm working distance on a Zeiss Sigma 500 (Zeiss for German). Particular attention was given to (i) the interfacial transition zone (ITZ) between HTS particles and the surrounding cement mortar, and (ii) the morphology of the internal voids within the slag itself.

## 3. Results

### 3.1. Stress-Wave Propagation Characteristics

**Figure 3:**
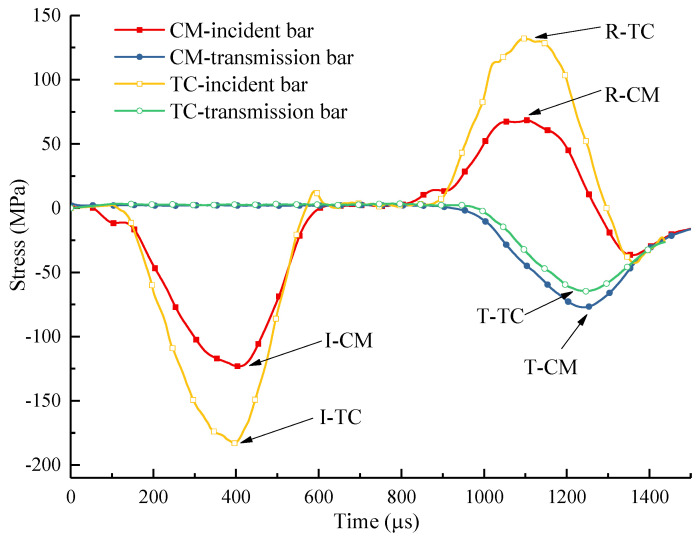
Stress wave values monitored in the incident bar and transmission bar of Specimen CM1 and TC1.

In Figure 3, I-TC, R-TC, and T-TC are the maximum values of incident stress wave, reflected stress wave, and transmitted stress wave of the TC group, respectively. I-CM, R-CM, and T-CM are the maximum values of incident stress wave, reflected stress wave, and transmitted stress wave in the CM group, respectively. After the calculation, αCM1, αCM2, and αCM3 are 62.72%, 60.71%, and 65.52%, respectively, and αTC1, αTC2, and αTC3 are 35.34%, 37.63%, and 36.25%, respectively. The results showed that the transmittance of the TC group decreased sharply, indicating that it had a good stress wave blocking effect.

**Figure 4:**
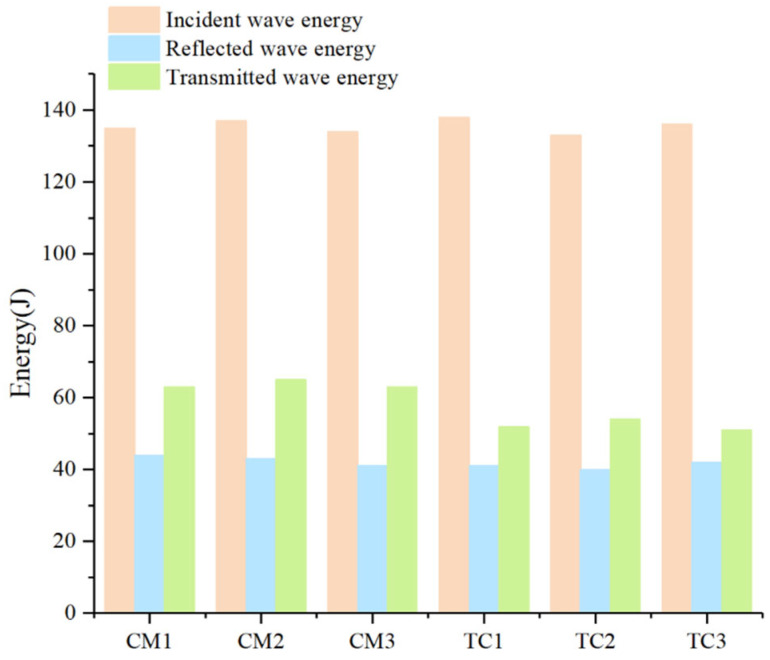
Time-dependent accumulated energy of the two materials.

The energies of the incident, reflected, and transmitted waves were subsequently quantified. To isolate strain-rate effects, the energy-absorption capacities of plain cement mortar and the high-titanium-slag concrete (HTS) were compared under identical loading rates. The temporal evolution of stress-wave energy is shown in Figure 4. After the calculation, βCM1, βCM2, and βCM3 are 20.74%, 21.17%, and 22.39%, respectively, and βTC1, βTC2, and βTC3 are 32.61%, 29.32%, and 31.62%, respectively. The average energy-attenuation coefficient of the HTS specimens exceeded that of the CM group by 9.7%.

**Figure 5:**
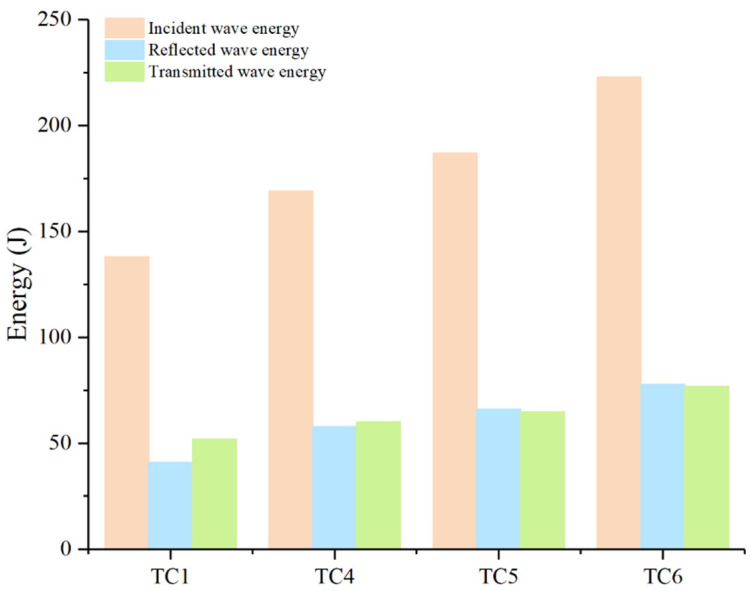
Time-dependent accumulated energy of the two materials under different loading rates.

Under dynamic loading, strain-rate effects exert a pronounced influence on a material’s mechanical response. A material can be regarded as stable under impact conditions only if it continues to meet its target performance across a range of loading rates. Accordingly, the energy-transfer characteristics of HTS specimens were evaluated at several strain-rate levels, as shown in Figure 5. The calculated energy-attenuation coefficients are β_TC1 = 32.61%, β_TC4 = 30.18%, β_TC5 = 29.95%, and β_TC6 = 30.49%. These results reveal that, even as the loading rate increases, HTS maintains a nearly constant energy-absorption capability, confirming its stability and reliability under high-rate loading.

### 3.2. CT Scanning Results

**Figure 6:**
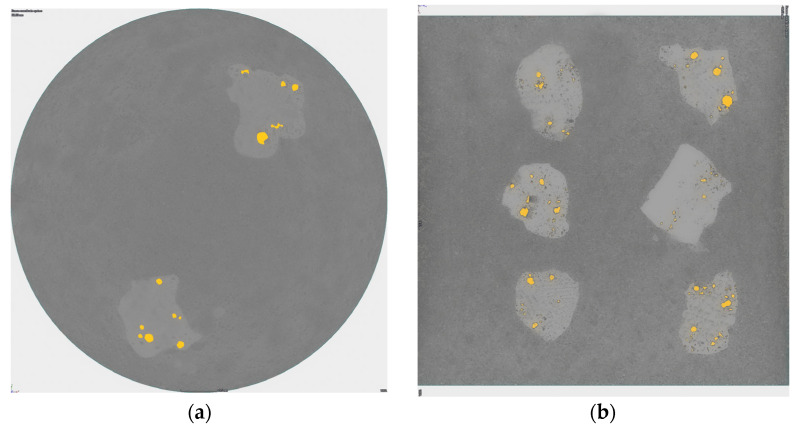
CT reconstruction of characteristic cross-sections of the HTC specimen: (**a**) top view; (**b**) side view.

Figure 6 shows reconstructed CT slices of the HTS specimen along two characteristic planes. The yellow-highlighted regions correspond to intra-aggregate cavities within the high-titanium slag (HTS), with diameters of 1–3 mm. Pore-size thresholding confirms that the cement mortar is essentially free of detectable air voids, demonstrating that incorporating porous HTS aggregates does not compromise its density. These quasi-elliptical cavities introduce multiple impedance interfaces during stress-wave propagation, thereby promoting wave scattering and energy dissipation.

### 3.3. SEM Scanning Results

**Figure 7:**
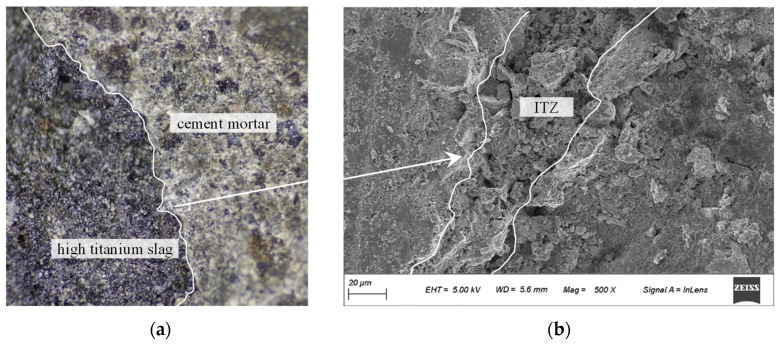
Interface amplification diagram of high-titanium slag and cement mortar. (**a**) Optical micrographs. (**b**) SEM micrographs.

Static compressive strength was tested to determine the material difference. The 28-day compressive strengths of specimens CM1, CM2, and CM3 are 38.5 MPa, 36.5 MPa, and 39.2 MPa; at the same time, the compressive strength of specimens TC1, TC2, and TC3 is 39.3 MPa, 40.1 MPa, and 38.6 MPa. This indicates that the static compressive strength of the two materials is significantly close. Based on the crushed specimen TC1, optical microscopy and scanning electron microscopy (SEM) were used to observe microscopic phenomena, as shown in Figure 6. The two media show a clear interface, as shown in Figure 7a, but the two media are closely connected to form a very dense interface transition zone (ITZ), as shown in Figure 7b. The sufficient hydration reaction is carried out, which is also the core reason why the TC group can maintain the static compressive strength, although the slag particles are porous. By leveraging elemental probing, the ITZ where HTS and cement paste bonded was identified, enabling clear observation of the aggregate, ITZ, and cement paste simultaneously. By calculating the area of the ITZ and dividing it by the contact particle boundary length, the average thickness of the ITZ measures approximately 26.3 μm. High titanium slag (HTS) aggregate particles contain a certain proportion of glass phase, which exhibits micro-volcanic ash activity. In the alkaline environment generated by cement hydration, the glass phase on the surface layer of the aggregate is activated, leading to the continuous dissolution of ions such as Ca^2+^, Al^3+^, Mg^2+^, and Si^4+^ [30]. These reaction products fill the interfacial gaps between the aggregate surface and the cement matrix, enhancing the interfacial density and ultimately improving the mechanical properties of the concrete.

The high-titanium slag (HTS) aggregates enhance the energy-absorption capacity of the concrete by promoting stress-wave scattering and attenuation through their internal porous structures and the formation of dense interfacial zones with the surrounding matrix—all while maintaining the overall mechanical strength of the material.

## 4. Numerical Simulation Analysis

**Figure 8:**
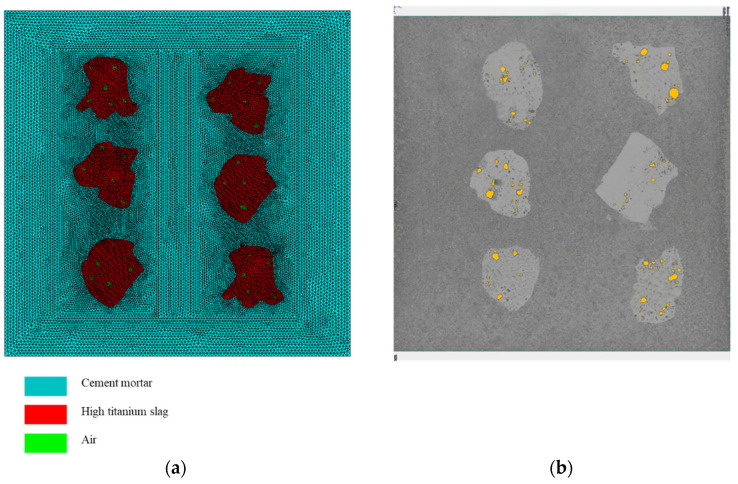
Numerical model and mesh discretization of HTS concrete. (**a**) Grid division of HTS samples. (**b**) The results of CT scan image.

To further verify the role of pore structures in absorbing stress waves within high-titanium slag (HTS) concrete and to elucidate the underlying mechanism, dynamic simulations were conducted using the finite difference software Autodyn (V19.2). As illustrated in Figure 8, a two-dimensional cross-sectional model was constructed based on the actual specimen, with dimensions of 100 mm × 100 mm. During meshing, the geometry of the HTS aggregates was reconstructed as faithfully as possible from CT data. Mesh refinement was applied locally within the HTS particles and their surrounding regions to enhance numerical accuracy. The model consists of 47,292 independent triangular elements.

In the dynamic simulation, an incident compressive stress wave was applied to the left boundary, while a transmissive boundary condition was imposed on the right to eliminate wave reflections. To isolate the energy dissipation caused solely by the internal cavity structures of the HTS aggregates, the amplitude of the applied stress wave was carefully controlled to ensure that the specimen remained within the elastic regime, thereby avoiding any wave-interfering damage or cracking. Both the cement mortar and the HTS aggregates were modeled as linear-elastic materials, and the internal pores were described using an ideal gas equation of state.

**Figure 9:**
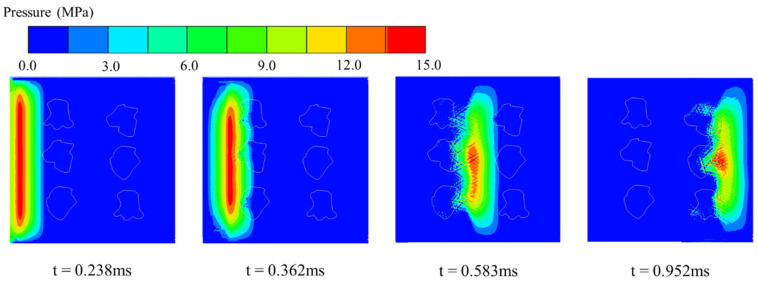
Stress-wave propagation process in HTC concrete.

Figure 9 illustrates the evolution of stress-wave propagation within the HTS concrete specimen. At t = 0.238 ms, the incident wave has just entered the specimen, maintaining a nearly planar front with a peak pressure of approximately 15 MPa. By t = 0.362 ms, the leading edge of the wavefront encounters the first row of HTS aggregates, forming a concave pressure trough on the incident side of the particles, with the peak dropping to about 13 MPa. At t = 0.582 ms, the wave has traversed the first HTS aggregate band, and the trailing portion becomes mottled in appearance, with the central peak reduced to around 10 MPa. Finally, at t = 0.952 ms, the wave exits the second HTS aggregate group, and the peak further attenuates to roughly 6 MPa—representing a 60% reduction compared to the initial incident value. This progression confirms that millimeter-scale pore structures within the aggregates can effectively scatter stress-wave components across multiple frequency bands and extend the temporal load window, thereby promoting substantial energy dissipation and redistribution.

**Figure 10:**
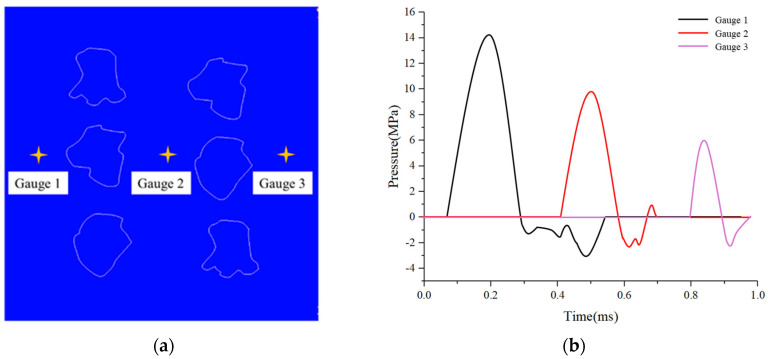
Pressure histories at characteristic monitoring points in the numerical model (**a**) schematic diagram of point locations; (**b**) time histories of pressure at different monitoring points.

Since the SHPB testing system cannot directly capture the internal evolution of stress waves within the specimen, three representative monitoring points were embedded in the numerical model, as shown in Figure 10a, to record the internal stress histories. The attenuation results are presented in Figure 10b. At Monitoring Point 1, the peak compressive stress reaches 14.7 MPa with a pulse width of approximately 0.15 ms. At Point 2, the peak decreases to 9.7 MPa and the pulse broadens to around 0.19 ms. At Point 3, the peak stress further drops to 6.2 MPa, with a pulse width of about 0.22 ms. These results indicate that the hierarchical pore structure of the HTS aggregates effectively attenuates the stress-wave amplitude and simultaneously extends its temporal duration. Notably, a small tensile stress phase is observed at Point 2, suggesting that secondary wave reflections occur between adjacent aggregates, contributing to additional energy dissipation within the material.

**Figure 11:**
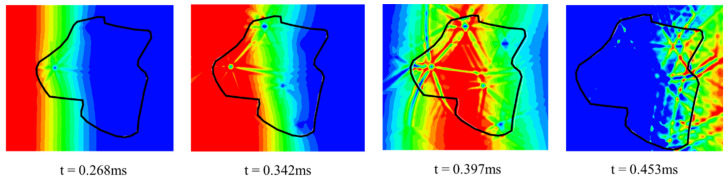
Localized amplification characteristics of stress wave propagation in high-titanium slag aggregate.

Figure 11 presents a magnified view of the stress-wave propagation within a single high-titanium slag (HTS) aggregate. Upon initial contact with the aggregate surface, the incident wave encounters pore structures with extremely low acoustic impedance, resulting in near-total reflection and the formation of radial backscattered waves around the cavities. As the wave penetrates deeper into the aggregate and interacts with multiple internal pores, it undergoes repeated reflections and diffractions, producing a network of intersecting high-pressure bands. These eventually evolve into grid-like tensile zones within the aggregate. This complex transmission process significantly attenuates the incoming stress wave, thereby enhancing the material’s capacity to absorb dynamic energy.

**Figure 12:**
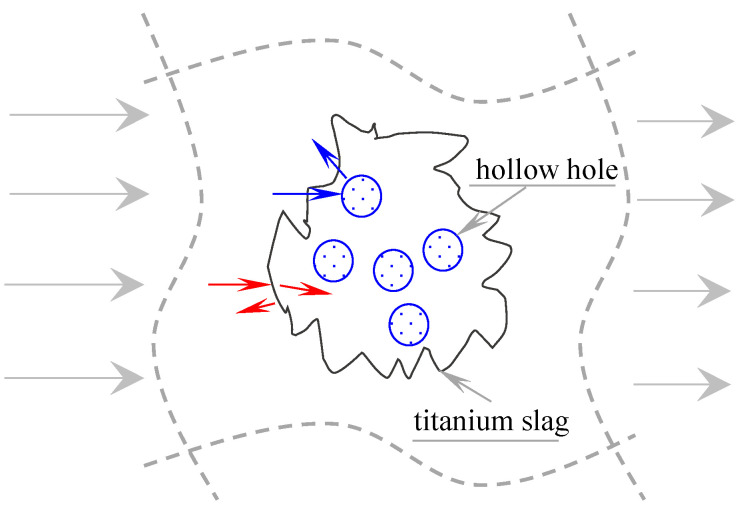
Schematic diagram of attenuation mechanism of stress wave.

When the stress wave propagates in the specimen, it is first reflected and transmitted on the surface of the slag after encountering the slag, as shown by the red arrow in Figure 12. However, the wave impedance difference between the two solid materials is very small, and the ITZ is dense, which will not form an obvious reflection effect, and the energy can be transmitted into the slag particles. However, when the stress wave encounters a hole, because it is transmitted from the solid material to the gaseous material, the stress wave is completely reflected. As shown in the blue arrow in Figure 12, the stress wave cannot propagate through the empty hole, which is the fundamental reason for the sharp decrease in the transmittance of the TC group. This is also the fundamental advantage of the high-titanium slag concrete as a protective material for underground engineering.

## 5. Conclusions

A novel high-titanium slag (HTS) concrete with multiscale pores was designed to address the critical challenge of balancing stress wave attenuation and structural integrity in underground defense systems. By integrating large-particle porous HTS aggregates into cement mortar, the material retained comparable static compressive strength to conventional concrete while achieving a 23.08%~30.18% reduction in stress wave transmissivity. The multiscale pore architecture induced stress wave scattering and interfacial reflections at gas–solid boundaries, effectively dissipating energy. Microscopic analysis revealed a dense interface transition zone (ITZ) between HTS aggregates and the cement mortar, ensuring mechanical coherence. Split Hopkinson Pressure Bar (SHPB) tests confirmed that wave attenuation stemmed primarily from pore-driven reflections rather than impedance mismatch. AUTODYN enables effective analysis of the stress-wave absorption mechanisms in HTS concrete. The intrinsic pore structure of the high-titanium slag aggregates can significantly scatter stress-wave energy and effectively extend the load duration while reducing the peak amplitude, thereby enhancing the material’s resistance to dynamic loading. This work demonstrates a scalable strategy for engineering blast-resistant materials that synergize dynamic energy dissipation with structural robustness, offering significant potential for enhancing the protective capacity of underground infrastructure against high-impact threats.

1. Under identical loading-rate impacts, the TC group’s energy-attenuation coefficient β averages 31.18%, versus 21.43% for the CM group—an increase of 9.75 percentage points, confirming that HTS absorbs stress-wave energy.
2. As the loading rate increases stepwise, the TC group’s β fluctuates between 29.95% and 32.61%, demonstrating stability under dynamic impact.
3. CT and SEM confirm 1–3 mm intra-HTS pores and a dense interfacial transition zone (ITZ). Numerical results further show that after traversing two bands of HTS particles, the stress-wave peak decreases from 15 MPa to 6 MPa, while the pulse width broadens from 0.15 ms to 0.22 ms—indicating that reflections at solid–gas interfaces and multiple scattering in HTS drive amplitude attenuation and, consequently, energy dispersion.

## Figures and Tables

**Table 1:** Chemical composition of HTS.

Composition	CaO	SiO_2_	TiO_2_	Al_2_O_3_	MgO	Fe_2_O_3_	SO_3_	MnO	K_2_O
Content	27.92	23.63	23.63	13.30	7.76	1.53	1.15	0.75	0.50

## Data Availability

The original contributions presented in this study are included in the article. Further inquiries can be directed to the corresponding authors.
